# Comparison of the Pathogenic Potential of *Campylobacter jejuni*, *C. upsaliensis* and *C. helveticus* and Limitations of Using Larvae of *Galleria mellonella* as an Infection Model

**DOI:** 10.3390/pathogens9090713

**Published:** 2020-08-29

**Authors:** Krunoslav Bojanić, Els Acke, Wendi D. Roe, Jonathan C. Marshall, Angela J. Cornelius, Patrick J. Biggs, Anne C. Midwinter

**Affiliations:** 1 ^*m*^EpiLab, Infectious Disease Research Centre, School of Veterinary Science, Massey University, Palmerston North 4410, New Zealand; J.C.marshall@massey.ac.nz (J.C.M.); p.biggs@massey.ac.nz (P.J.B.); A.C.Midwinter@massey.ac.nz (A.C.M.); 2 Klinik für Kleintiere, Veterinärmedizinische Fakultät der Universität Leipzig, 04103 Leipzig, Germany; els.acke@kleintierklinik.uni-leipzig.de; 3 Department of Pathology, School of Veterinary Science, Massey University, Palmerston North 4410, New Zealand; W.D.Roe@massey.ac.nz; 4 Institute of Environmental Science and Research Limited, Christchurch 8540, New Zealand; angela.cornelius@esr.cri.nz

**Keywords:** *Galleria mellonella*, pathogenicity, virulence, *Campylobacter*, dose response, emerging pathogens, survival

## Abstract

*Campylobacter* enteritis in humans is primarily associated with *C. jejuni/coli* infection. Other species cause campylobacteriosis relatively infrequently; while this could be attributed to bias in diagnostic methods, the pathogenicity of non-*jejuni/coli Campylobacter* spp. such as *C. upsaliensis* and *C. helveticus* (isolated from dogs and cats) is uncertain. *Galleria mellonella* larvae are suitable models of the mammalian innate immune system and have been applied to *C. jejuni* studies. This study compared the pathogenicity of *C. jejuni*, *C. upsaliensis*, and *C. helveticus* isolates. Larvae inoculated with either *C. upsaliensis* or *C. helveticus* showed significantly higher survival than those inoculated with *C. jejuni*. All three *Campylobacter* species induced indistinguishable histopathological changes in the larvae. *C. jejuni* could be isolated from inoculated larvae up to eight days post-inoculation whereas *C. upsaliensis* and *C. helveticus* could only be isolated in the first two days. There was a significant variation in the hazard rate between batches of larvae, in *Campylobacter* strains, and in biological replicates as random effects, and in species and bacterial dose as fixed effects. The *Galleria* model is applicable to other *Campylobacter* spp. as well as *C. jejuni,* but may be subject to significant variation with all *Campylobacter* species. While *C. upsaliensis* and *C. helveticus* cannot be considered non-pathogenic, they are significantly less pathogenic than *C. jejuni*.

## 1. Introduction

*Campylobacter*iosis is one the most common bacterial gastrointestinal diseases in people worldwide and has mostly been associated with *C. jejuni* and *C. coli* infection [1]. The disease is predominantly foodborne, especially through contaminated poultry meat, but as *Campylobacter* spp. is commonly isolated from the intestinal tract of many animals, direct contact with animals and with contaminated water and environments are also important transmission routes of infection [1]. Diagnostic methods are commonly optimised for the detection of *C. jejuni/coli*; hence, many other *Campylobacter* spp. species are considered as under-reported, although they are implicated as human pathogens, and are often referred to as ‘emerging’ pathogens [2,3]. *C. upsaliensis* and *C. helveticus* are the most common *Campylobacter* spp. reported in dogs and cats, where they are frequently detected with considerable prevalence rates that reach up to 63% [3,4], whereas they are rarely reported, with low prevalence rates (~1–2%) in other sources [5,6,7,8,9]. *C. upsaliensis* is one of the leading emerging *Campylobacter* pathogens, as several studies worldwide have reported higher isolation rates from human campylobacteriosis cases than the rate of *C. coli* when suitable culture methods are applied [10]. Conversely, no or few occurrences have also been reported with suitable methods [11,12,13] which could be affected by geographical, seasonal and environmental exposure differences between study populations. *C. helveticus* closely resembles *C. upsaliensis* [14] but has only been reported in one human study previously [15]. Considering that the two species are common in pet animals but infrequent and disparate in humans, their pathogenic potential remains uncertain.

Mechanisms of pathogenesis of *Campylobacter* spp. can be investigated by various approaches such as in vivo research using “complete” or “partial” animal models of disease that reproduce intestinal disease in many or only a few aspects of pathogenesis and virulence (primates, rodents, chickens, etc.), or ex vivo approaches such as eukaryotic cell cultures, and molecular biology tools which are necessarily “partial” models of disease because only particular aspects of disease stages (e.g., colonisation, adhesion, invasion, toxigenesis, or roles of genes using mutant construction) can be evaluated [16]. Invertebrates may also be used as an infection model due to a high degree of functional and structural homology with the mammalian innate immune system [17]. The larvae of the greater wax moth, *Galleria mellonella*, have been described as a model for many bacterial pathogens, including species of *Acinetobacter, Escherichia, Enterococcus*, and *Pseudomonas* [18,19]. Larvae were also described as an infection model for *C. jejuni* for the evaluation of the role of selected genes in the mortality of larvae [20]. In that study, mutant strains of *C. jejuni* lacking defined virulence factors showed an increased survival of larvae compared to the respective wild types. Subsequently, the histological changes and survival of *C. jejuni* in the larvae were described and used to evaluate differences in virulence between genotypes of *C. jejuni* according to the multi-locus sequence-typing scheme [21]. That study showed extensive histological changes in larvae upon inoculation of *C. jejuni*, and intracellular survival of *C. jejuni* within larval haemocytes.

The aim of this study was to use *Galleria mellonella* larvae as an infection model for the comparison of the pathogenic potential between *Campylobacter* species. *C. upsaliensis* and *C. helveticus* were selected as the emerging pathogens to which humans are likely to be frequently exposed from contact with pets and were compared to the established pathogen *C. jejuni*. The survival of larvae inoculated with viable bacteria and temperature-inactivated cells and cellular components, histopathological changes, cultures of larval haemolymph and haemocytes, and dose-dependent survival analyses were used as comparative features of larval infection between *Campylobacter* species.

## 2. Results

**Figure 1:**
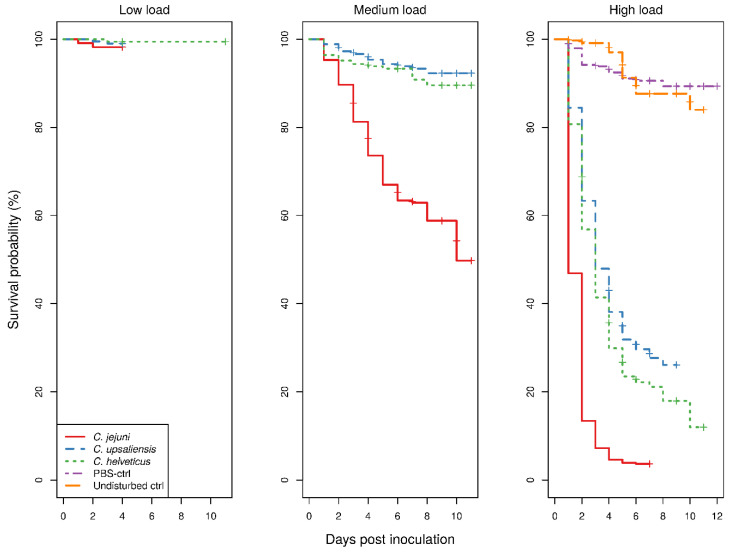
Kaplan–Meier survival curves of 4273 larvae inoculated with three loads (~10^4^, 10^6^, and 10^8^ CFU/mL) of *Campylobacter* spp. and of control (280 each of phosphate-buffered saline (PBS)-inoculated and undisturbed) larvae groups. The survival curves are a summation of 34 strains of *C. jejuni*, 22 strains of *C. upsaliensis*, and 13 strains of *C. helveticus* with each bacterial strain tested with two to four biological replicates and each tested in technical duplicates of sets of 10–15 larvae. In the low load only 12 strains of both *C. jejuni* and *C. upsaliensis,* and 10 strains of *C. helveticus* were evaluated. There were 1861 larvae used with high loads (821 *C. jejuni,* 630 *C. upsaliensis* and 410 *C. helveticus* larvae), 1802 with medium loads (785 *C. jejuni*, 625 *C. upsaliensis* and 392 *C. helveticus* larvae), and 610 with low loads (225 *C. jejuni*, 205 *C. upsaliensis* and 180 *C. helveticus* larvae).The control larvae groups of the same experimental run are applicable to all loads prepared, but are shown only in one subplot for clarity. All assays were performed in a H_2_-enriched microaerobic atmosphere (H_2_-MA; 82% N_2_, 10% CO_2_, 5% H_2_, 3% O_2_) at 37 °C.

The Kaplan–Meier survival curves for larvae showed a clear dose-dependent survival for all *Campylobacter* spp. (Figure 1).

With low bacterial doses, there were too few deaths recorded which precluded comparison of any of the treatment groups. With medium doses, the survival of *C. jejuni-*inoculated larvae was significantly lower than that of both *C. upsaliensis-* or *C. helveticus-*inoculated larvae (*p* < 0.001), the latter two not being significantly different to each other. In contrast, with high doses, the survival of larvae was significantly different between all *Campylobacter* spp. (*p* < 0.001). The survival of control groups showed no difference with low bacterial doses of any *Campylobacter* spp.-inoculated larvae. The uninfected larvae and larvae infected with low *Campylobacter* doses never exhibited macroscopic melanisation, although reduced responses to physical stimuli were observed in the infected larvae only. Signs of morbidity in infected larvae with medium and high doses were always present. With the medium bacterial dose, only the survival of *C. jejuni*-inoculated larvae was significantly different from that of the control groups (*p* < 0.001), whereas with the high bacterial dose all *Campylobacter* spp.-inoculated larvae had a significantly different survival from the control groups (*p* < 0.001). The survival of phosphate-buffered saline (PBS)-inoculated and undisturbed larvae was not significantly different. The results of the assays using temperature-inactivated bacteria or cellular components and different incubating conditions are available in the Supplementary Materials.

**Figure 2:**
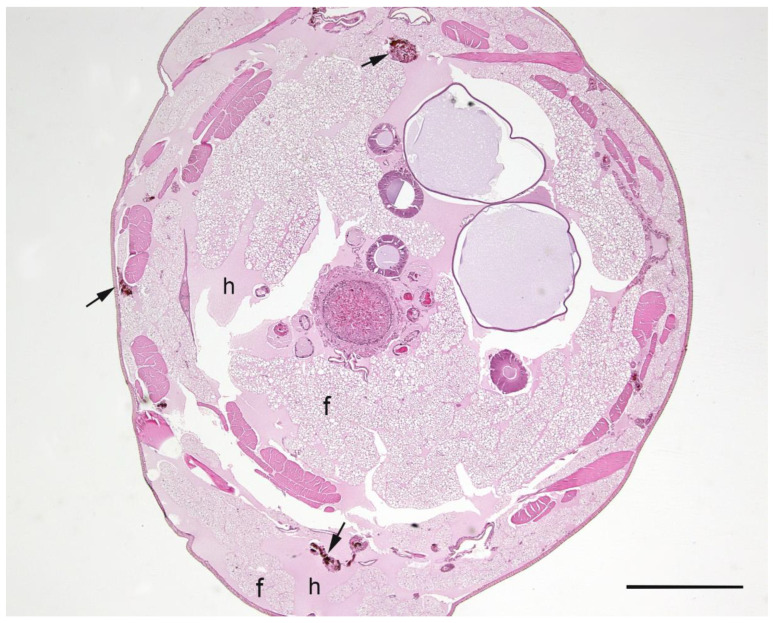
Haematoxylin and eosin stained cross section of *Galleria mellonella* larva inoculated with *Campylobacter helveticus* that was observed dead on the second day of observation. Structures are identified as follows: fat bodies (f); haemolymph (h). Pigmented clusters of haemocytes are indicated by arrows. Bar = 1 mm.

**Figure 3:**
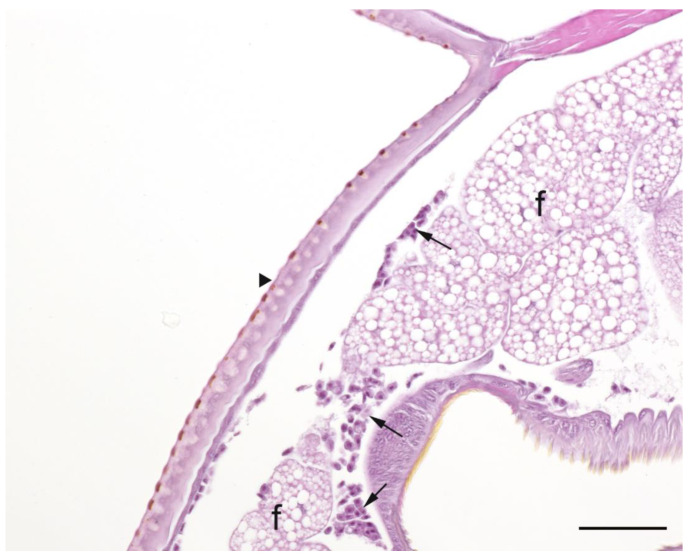
Haematoxylin and eosin stained section of subcuticular region of *Galleria mellonella* larva, inoculated with *Campylobacter* *upsaliensis* that was observed alive on the third day of observation showing cuticle (arrowhead), fat bodies (f), and clusters of non-pigmented haemocytes. Bar = 25 µm.

**Figure 4:**
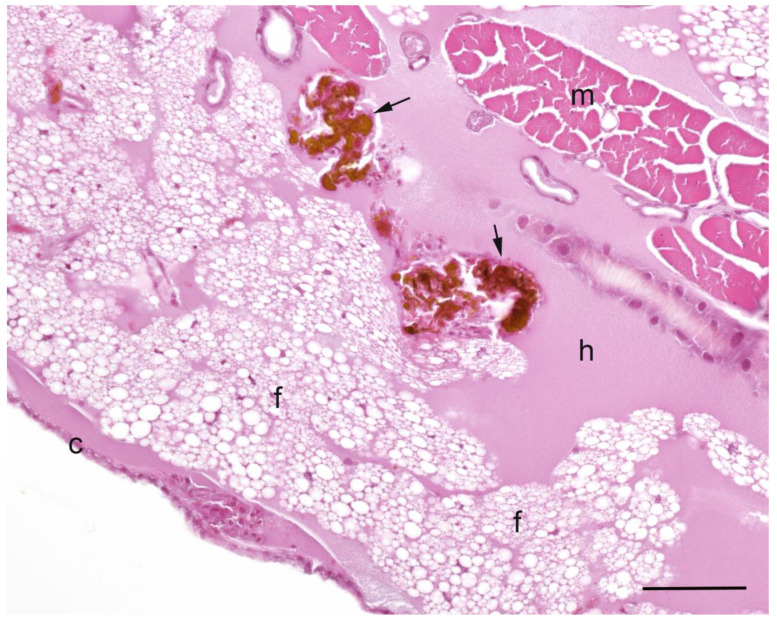
Haematoxylin and eosin stained section of *Galleria mellonella* larva inoculated with *Campylobacter upsaliensis* that was observed dead on the second day of observation, showing densely pigmented clusters (nodules) of haemocytes (arrows) within haemolymph (h) adjacent to fat bodies (f). Other structures are indicated as follows: muscle (m); cuticle (c). Bar = 25 µm.

**Figure 5:**
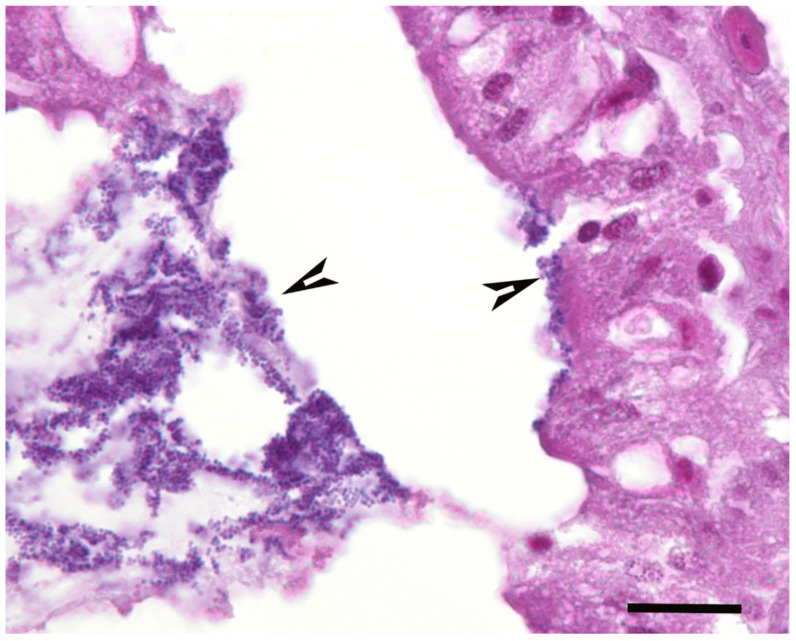
Haematoxylin and eosin stained section of *Galleria mellonella* larva inoculated with phosphate-buffered saline that was observed alive on the third day of observation showing bacteria within the gastrointestinal tract lining the mucosal surface (right arrowhead) and free within the lumen (left arrowhead). Bar = 25 µm.

A summary of the histopathological scores of infected and uninfected larvae is presented in Table 1. The gut tissues in both infected and uninfected larvae could not be evaluated due to heavy autolysis. Overall, the scores of fat bodies and haemolymph between infected and uninfected larvae were similar, but the two features showed large variations between larvae from the same experimental runs, and thus were not statistically analysed. The scores of haemocytes were not significantly different between the infected and uninfected larvae and between *Campylobacter* spp. within infected larvae. However, only the infected larvae exhibited pigmentation in haemocytes’ cytoplasm and pigment deposition in tissues (fat bodies, muscle, epithelial cells, haemolymph and gut lumen) and nodules (Figure 2, Figure 3, Figure 4 and Figure 5). The overall pigment scores were significantly higher in infected than in uninfected larvae (*p* < 0.01) but showed no difference between *Campylobacter* spp. within the infected larvae. The pigment scores in haemocytes, nodules and tissues between *Campylobacter* spp. within infected larvae were not significantly different. Nodules were only observed in the infected larvae but with no difference between *Campylobacter* spp. within infected larvae. Coccoid bacteria were almost always observed in infected larvae, but filamentous rods and cocci were also noted, though infrequently, in both infected and uninfected larvae. Scores for the abundance of bacteria were significantly higher in the infected than in the uninfected larvae (*p* < 0.001) but not between *Campylobacter* spp. within the infected larvae. Within the infected larvae, bacterial scores had a significant positive correlation with nodule scores (*rho* = 0.45, *p* < 0.01) and pigment scores (*rho =* 0.41, *p* = 0.01). Similarly, bacterial scores had a significant positive correlation (*rho* = 0.36, *p* = 0.04) with bacterial doses of inocula in infected larvae.

The isolation of *Campylobacter* spp. from the haemolymph and haemocytes of larvae was attempted on days one, two, three, five and eight post-inoculation. The isolation was successful with all of the *Campylobacter* spp. from the haemolymph and from the haemocytes only with *C. jejuni* but never from uninfected control larvae. The isolation of *C. upsaliensis* and *C. helveticus* from the haemolymph was only successful in the first two days post-inoculation while *C. jejuni* was successfully isolated, including from the haemocytes, up to eight days post-inoculation.

Cox proportional hazard (CoxPH) mixed effects regression analysis showed the larvae batch shipment, and a nested structure of strain, bacterial dose, and biological and technical replicates were significant random effects on the survival of larvae. The final model showed a significant variation between species, as a fixed effect, with a hazard rate for *C. upsaliensis* and *C. helveticus* of 21% (95%CI 14–30%, *p* < 0.001) and 34% (95%CI 22–52%, *p* < 0.001) respectively, of the hazard rate of *C. jejuni*. The hazard rate of *C. upsaliensis* was 61% (95%CI 39–95%, *p* = 0.03) of the hazard rate of *C. helveticus*. An increase of one log unit from the average bacterial dose (~6.5 × 10^6^ CFU/mL +/−3 × 10^5^ standard error of the mean) increased the hazard rate by 4.6 times (95%CI 4.1–5.2, *p* < 0.001) as an independent fixed effect. The estimated standard deviation of the random effects was used to present the variability within 95% of the population from the population average estimates. The final CoxPH model showed the variation in the hazard rate of larval survival of batch shipments to be in the range of 61–165% of the average batch estimate. For the nested structure of strains and replicates, the variation in the hazard rate of bacterial strains was in the range of 81–141% of the average strain estimate, for biological replicates it was in the range of 77–135% of the average biological replicate estimate, extending to a 41–300% variation in the average estimate of bacterial dose used and, for the technical replicates, to 95–107% of the average technical replicate estimate. Importantly, only the technical replicates did not significantly contribute to the final model but were kept in order to quantify the effect of a commonly employed experimental procedure. The mixed effects CoxPH model for control groups showed no significant difference between the hazard rate of undisturbed and PBS-inoculated larvae. The random effect of the batch shipments on the survival of control larvae groups ranged from 25 to 319%, and for technical replicates it range from 76 to 146% of the average estimate.

## 3. Discussion

To be useful as a descriptor of pathogenic features, the survival/death of inoculated larvae must be sufficiently contrasting between groups to allow differentiation. This differentiation is not possible between groups inoculated with lower doses of *Campylobacter*; however, it is distinct when a higher dose (10^8^ CFU/mL) is used (Figure 1). The difference in survival curves between species in the larvae model is suggestive of different pathogenicities, i.e., higher survival with lower pathogenicity and vice versa. However, histopathological findings were not different between the *Campylobacter* species. The histopathological findings in this study correlate well with the results of a study by Senior et al., investigating *C. jejuni* infection in larvae [21]. In that study, loss of integrity of the gut wall, activated and apoptotic haemocytes, pigment and nodule formations (observed macroscopically as melanisation) throughout the tissues and haemocoel were recorded in the infected larvae but not in the control larvae, as in the present study. These consistent histological changes in *Campylobacter* spp.-inoculated larvae and the clear differentiation from the changes in uninfected controls indicate that larvae as an infection model are also suitable for *C. upsaliensis* and *C. helveticus*. The presumption that coccoid bacteria observed in larval tissue sections are *Campylobacter* bacteria is supported by the successful isolation of *Campylobacter* spp. from larval tissues and the significant positive correlation of the bacterial scores with pigment and nodules scores and with the bacterial doses of the inocula used. The study by Senior et al. [21] documented the survival of *C. jejuni* in larval haemocytes using green fluorescent protein-tagged cells and found that the number of intracellular *C. jejuni* bacteria increases at 24 h compared to 4 h post-inoculation, suggesting that *C. jejuni* can not only survive but also replicate in larvae cells. This is in line with the results of haemolymph and haemocyte cultures of *C. jejuni*-inoculated larvae being successful up to eight days post-inoculation in the present study. In contrast to the histological findings, the present study showed many differences between *Campylobacter* spp. in the survival of larvae, the critical importance of the evaluation of dose dependency for a comparison of the three *Campylobacter* spp., and batches, strains of *Campylobacter* species, and biological and technical replicates as sources of variability.

To use the survival of larvae as an endpoint for pathogenic features, the dose requires a sufficiently different survival of larvae to allow a relative comparison with control larvae groups or other treatment group(s) [22]. The divergent survival of larvae inoculated with different *Campylobacter* spp. may not be observable below a certain dose, yet may be observable for all species at higher doses and, depending on the dose used, the difference from the survival of control larvae may vary (Figure 1). However, the dose used must provide a sufficient distinction from control groups, as otherwise the experiment would be deemed invalid. The mortality of control groups has no strict criteria, but, generally, it is expected to be as low as possible and not more than 10–20%. This is in line with the results of the CoxPH model, which showed no significant dose and species interaction, indicating that the effect of dose is the same across the *Campylobacter* spp. examined. The dose-dependent survival of *Galleria mellonella* larvae has been documented for many pathogens and differences can be as extreme as up to several thousand-fold within species and within bacterial genera [19]. Several studies of *C. jejuni* have reported strain-related variations in the survival of larvae. Excluding the data for mutant isolates, the ranges in the survival of larvae were reported as 10–20% [23], 25–43% [20], and 3–36% [24] in studies comparing less than five strains, and 50–100% in a study comparing 67 strains [21]. These studies were matched by the dose used (which corresponds to the medium dose in this study), and all evaluated the survival at 24 h except for Humphrey et al. [24], who evaluated larvae at 48 h post-inoculation. The overall survival of larvae in this study is much higher than in previous studies, except for the study by Senior et al. [21] for the corresponding time of observation and dose, which could be explained by, but not limited to, different suppliers of larvae and the different bacterial strains used. This is further supported by the findings of the significant random effects of batches, strains and biological replicates in the current study, whose magnitudes show that independent experimental studies may return similar survival rates despite a 100-fold difference in bacterial doses of the same strain, as well as a disparate survival rate with the same dose and species/strain used. The small variation estimated for the technical replicates and their insignificant contribution to the regression model show that consistent, reproducible results were obtained within the experimental runs. This suggests that internal validity for an observed difference is achievable in controlled experiments.

*Galleria mellonella* is a relatively new invertebrate model compared to *Drosophila melanogaster*, or *Caenorhabditis elegans*, which have stock centres and community databases such as Flybase and WormBase, respectively [25]. The lack of sources of genetically defined larvae strains and reference populations, different breeding and maintenance practices between suppliers, and maintenance and non-standardised experimental procedures between research laboratories, have been frequently raised as limitations of experimental comparability [19,22,25]. Since, in this study, only one supplier was used, the significant batch effect could be expected to be even larger for differences between suppliers than within suppliers, which is a significant issue when comparing studies. The CoxPH model showed some violations of the proportionality of the hazard rates, which caution us that the magnitude of the estimates of species differences is not constant over the observation period. The violations were not severe enough to invalidate the model, as no inverse relationships occurred, as shown by the fact that there was no crossing of the Kaplan-Meier survival curves (Figure 1). However, this finding raises questions about the choice of observation time for comparisons to be made. All of the previous studies of *C. jejuni* in larvae with observations at 24 h had a 100% survival among control groups and survival was over 98% at 48 h, the longest observation reported [24]. This is in line with the current study, as the ~10% mortality of control larvae groups was reached around the fifth day of observation and remained constant until at least 10 days (Figure 1). This relatively low mortality rate of the control groups in the present study shows that the observation period can be prolonged and that more information about infection dynamics can be revealed in inoculated larvae.

The assays with altered environmental incubating conditions (see Supplementary Materials) in this study showed improved survival for larvae infected with all *Campylobacter* spp. as the conditions become less favourable for *Campylobacter* growth in vitro, with the control groups not being adversely affected. A seminal study of *Campylobacter* in the larvae model reported the death of all infected larvae at 25 °C and of control larvae at 42 °C but not at 37 °C, suggesting the model to be suitable for mammalian but not avian gut temperatures; however, the exact atmospheric conditions of the larval experiments were not provided [20]. Multiple other studies used an incubation temperature of 37 °C; however, only one reported atmospheric conditions (in that study, room temperature and an aerobic atmosphere were used) [20,21,26,27,28,29,30]. This study showed that an H_2_-enriched microaerobic atmosphere (H_2_-MA), which more closely mimics gut conditions than an aerobic environment, is also suitable for the *Galleria* model and indicates that H_2_-requiring *Campylobacter* spp. of clinical importance [3] could be tested as well. However, while the extent of changes inside the larvae due to the outside environment was not evaluated herein, more importantly, due to the survival of infected larvae being affected by the incubation atmospheric conditions, we recommend their inclusion in the methods of future studies as an important variable. The influence of environmental variables on the immune responses of larvae and the resulting limitations of their use as an animal model is still largely unknown. Thus, the increased survival of *Campylobacter* spp.-inoculated larvae incubated in decreasingly favourable incubating conditions for *Campylobacter* spp. could be due to an altered immune response on the host side, decreased numbers of viable bacteria in the larvae, or environment-induced changes in metabolic/virulence capacities of *Campylobacter* and should be investigated further to understand the infection dynamics in the larvae model. Similarly, the results of assays with inactivated bacteria indicate that more detailed studies of cellular components would be beneficial to evaluate their role in the morbidity/mortality of larvae. Melanisation of larvae in these assays was evident, which is a sign of the activation of the immune system that may also cause substantial damage to the host and lead to death [31]. Interestingly, the survival of larvae inoculated with inactivated bacteria was not different between *Campylobacter* spp. in contrast to survival following inoculation with viable bacteria. This pattern suggests that the metabolic activity of viable *C. upsaliensis* and *C. helveticus* cells contributes less to the death of larvae compared to their cell components than the activity of *C. jejuni* cells does.

Insect haemocytes recognise pathogens and phagocytise foreign material in a similar manner to mammalian neutrophils, with the killing of ingested microbes by both cell types by the production of superoxide and by the release of enzymes in the process of degranulation [32]. While there are a variety of cellular and humoral immune mechanisms that may also play a role in larvae [17], the survival of *C. jejuni* in haemocytes is likely to be due to the possession of genes involved with oxidative and aerobic stress tolerance. Studies have shown that the MarR-like transcriptional regulators, *rrpA* and *rrpB*, are involved in the regulation of catalase (*katA*), alkyl hydroperoxide reductase (*ahpC*) and superoxide dismutase (*sodB*) genes. These genes are involved in the tolerance of oxidative and aerobic stresses, and mutants of these genes and their regulators were reported to be associated with the significantly increased survival of larvae compared to the wild type isolate, a pattern not seen for mutants of the peroxide-sensing regulator (*perR*), and *rrpA* and *rrpB* double mutants [33,34]. Phenotypically, *C. upsaliensis* and *C. helveticus* are catalase weakly positive [35] and negative [14], respectively. However, the higher survival of *C. upsaliensis*-inoculated than of *C. helveticus*-inoculated larvae in the present study suggests that more than just catalase activity is needed to explain their survival.

This study demonstrates the larvae model as suitable for the investigation of the pathogenicity of *C. upsaliensis* and *C. helveticus* by survival analyses, histopathological changes, and culture of larval haemolymph and haemocytes. Their pathogenic potential was lower than that of *C. jejuni*, the major pathogen of the genus. The lower pathogenicity of the former species is most likely attributable to the faster clearance of the bacteria from haemolymph and their inability to survive in haemocytes in contrast to *C. jejuni*. The survival of larvae was not significantly different between the three *Campylobacter* spp. when inoculated with inactivated cells or cellular material, confirming the difference in pathogenic potential being due to the metabolism of live, viable cells. Survival analyses also showed that longer periods of observation are feasible in the larvae model and that a large variability in hazard rates exists due to the effects of larvae batches, species strains, and biological but not technical replicates that emphasise the limitations of comparisons between assays, yet confirm the internal validity within experimental runs. Lastly, assays with different incubating conditions indicate that the larvae model could be extended to H_2_-dependent *Campylobacter* and other similar bacterial species.

## 4. Materials and Methods

### 4.1. Strains and Cultures

Overall, there were 34 strains of *C. jejuni*, 22 strains of *C. upsaliensis*, and 13 strains of *C. helveticus*. Isolates of *C. jejuni* were selected to include frequent, common and rarely occurring sequence types (STs) in humans from the ^m^EpiLab collection, which contains over 3500 isolates from the Manawatu Sentinel Site Surveillance project, a source attribution study using concurrent sampling of sick people, animals, food and the environment [36]. *C. upsaliensis* and *C. helveticus* isolates were of unknown genotypes and were selected from previous studies in dogs and cats [37,38], and other external sources. As the main aim was species comparison, many strains were used in order to capture diversity at the species level and exclude the potential bias of individual strains. Species type strains were also included, and the details of all isolates are presented in Table 2.

Isolates were recovered from 15% (weight/volume) glycerol (#G/0650/17, Fisher Scientific, Loughborough, UK) in nutrient broth (#CM0067, Oxoid, Basingstoke, UK) vials stored at −80 °C by plating on Columbia horse blood agar (#1085, Fort Richard, Auckland, New Zealand) incubated in a H_2_-MA (82% N_2_, 10% CO_2_, 5% H_2_, 3% O_2_) in an M85 variable atmosphere workstation (Don Whitley Scientific, Bingley, UK) at 37 °C. Species identification was previously confirmed using PCR targeting the *hipO* gene for *C. jejuni* [39] and the 16S rRNA gene for *C. upsaliensis* and *C. helveticus* [40].

### 4.2. Galleria Mellonella Assays

Fifth instar larvae were obtained from Biosuppliers (Auckland, New Zealand), kept (as delivered) in a mixture of wood chips and honeycomb at room temperature, and used within four days. Using manual restraint of the larvae, inocula of approximately 10 µL were injected into the haemocoel via the last left proleg using 31G insulin syringes (#328822, Becton Dickinson Co., Holdredge, NE, USA) on sets of 10–15 larvae. Upon inoculation, the larvae were kept in Petri dishes with a moist tissue and no food. Larvae inoculated with phosphate-buffered saline pH 7.3 (PBS-inoculated) and undisturbed larvae were used as controls for the experimental procedure and the incubating conditions, respectively, and all assays were performed in technical duplicates. Larvae were observed daily for up to 12 days with a minimum of five days of observation. The morbidity and mortality assessment consisted of evaluation of colour changes (melanisation), righting reflex and responses to physical stimuli. The absence of the righting reflex and responses to stimuli were considered signs of death (mortality), while melanisation and either reduced responses to stimuli or the absence of the righting reflex were considered as signs of morbidity. A subset of larvae was used to culture *Campylobacter* spp. from haemolymph and evaluate whether *Campylobacter* was associated with haemocytes over several days post inoculation. The whole larvae were ethanol (70% vol/vol) washed, flamed and then the bottom ~2–3 mm of the body was aseptically removed and the haemocoel drained into a sterile microcentrifuge tube and centrifuged at 200 *g* for 5 min. The haemolymph (supernatant) was transferred to another tube, plated on blood and cefoperazone amphotericin teicoplanin (CAT) agars (#1048, Fort Richard, Auckland, New Zealand) and cultured as above. The pelleted haemocytes were resuspended in 1 mL sterile Milli-Q^®^ water (Merck-Millipore, prepared in-house), pipetted up and down ten times to lyse the cells and thereafter cultured as the haemolymph [21].

In total, 5878 larvae obtained from seven different batches were used for inoculation with *C. jejuni* (2137 larvae), *C. upsaliensis* (1751), *C. helveticus* (1272), PBS (338), and Mueller–Hinton broth (10) (#3222, Fort Richard, Auckland, New Zealand) while 385 served as undisturbed controls. The main focus was the inoculation of larvae with viable bacteria using three 100-fold dilutions incubated in H_2_-MA at 37 °C. For the three different bacterial loads evaluated, there were 1861 larvae used with high loads (821 *C. jejuni,* 630 *C. upsaliensis* and 410 *C. helveticus* larvae), 1802 with medium loads (785 *C. jejuni*, 625 *C. upsaliensis* and 392 *C. helveticus* larvae), and 610 with low loads (225 *C. jejuni*, 205 *C. upsaliensis* and 180 *C. helveticus* larvae). All bacterial strains in each experimental run were tested with high and medium loads but not low loads. The low load was discontinued due to the insufficient mortality of the larvae inoculated with bacteria compared with those of control groups; only 12 strains of both *C. jejuni* and *C. upsaliensis*, and 10 strains of *C. helveticus* were tested with low loads. Each bacterial strain was tested in two to four biological replicates, from which dilutions were made for the bacterial loads with technical duplicates for each load. Both control larvae groups always had two technical duplicates. The incubating conditions for these primary assays performed in an H_2_-MA at 37 °C were considered an ‘optimal environment’ and a small subset of larvae was used for assays using the inactivated bacteria or cellular material, and different incubation environments. The environment was categorized in terms of its ability to support the growth of *Campylobacter* species. The use of room temperature and H_2_-MA, or aerobic atmosphere and 37 °C was considered a ‘partial environment’, whereas the use of room temperature and an aerobic environment was designated as an ‘adverse environment’. The assays in different environments were performed to evaluate the dependencies on temperature and atmosphere of *Campylobacter* bacteria to cause the infection and/or death of the larvae. Assays with temperature-inactivated cells and cellular components were performed to evaluate their effect on the survival of larvae. As these assays were not the primary aim of the study, the biological replicates were not evaluated and only high loads with technical duplicates were tested using two to four strains of each species. Assays with inactivated bacteria or cellular material were always performed in an ‘optimal environment’.

The heat-inactivation of bacteria was performed by heating the inocula at 100 °C for 10 min in sterile microcentrifuge tubes placed in a heat block. The suspension of heat-killed bacteria was briefly vortexed and used as a ‘whole-cell’ assay and an aliquot was transferred to a sterile microcentrifuge tube and centrifuged at 12,000 *g* for 3 min. The supernatant was pipetted into a new sterile microcentrifuge tube, briefly vortexed and used as a ‘heat-inactivated soluble’ assay, while the pelleted cellular material was re-suspended in one mL of PBS, briefly vortexed and used as a ‘heat-inactivated insoluble’ assay. The cold-inactivation of bacteria was performed by a triple freeze–thaw procedure between −80 °C and 42 °C in three cycles of 30 min duration. The suspensions of cold-killed whole-cell and soluble assays were performed as for the heat-killed assays. The ‘cold-inactivated insoluble’ assay was omitted due to a visible inhomogeneity of samples that could not be improved by vortexing. For the heat- and cold-inactivated assays the inactivation was checked by culturing as described above. For the secretory products assay, bacteria were subcultured into 6 mL of Mueller–Hinton broth (Fort Richard, Auckland, New Zealand) and incubated in H_2_-MA at 37 °C in airtight boxes at 200 rotations per minute until turbid (usually two days for *C. jejuni* and *C. upsaliensis* and three to four days for *C. helveticus*). The broth was centrifuged in the same way as for the heat-inactivation assay with the supernatant pipetted into a sterile microcentrifuge tube and used as the ‘secretory products’ assay, while control larvae were injected with sterile Mueller–Hinton broth.

### 4.3. Campylobacter Inocula

One-hundred and thirty-five inocula were prepared by suspending one to two whole agar plates’ growth of *Campylobacter* spp. in PBS followed by one or two 100-fold dilutions in PBS. For a quantification of bacterial concentrations, the inocula were serially diluted, spiral-plated (WASP, Don Whitley Scientific, Bingley, UK) on blood agar and incubated as above. Bacterial colony counts were performed manually and/or using an aCOLyte colony counter (Synbiosis, Cambridge, UK). Subsequently, 18 inocula were adjusted to 0.05 light transmittance at 590 nm using a turbidimeter (#3587, Biolog, Hayward, CA, USA) from which dilutions for the inoculation of larvae and the quantification of bacterial concentration were performed. These estimates of bacterial concentration were used for the subsequent 72 inocula with the turbidity adjusted accordingly because colony counting was not performed further.

### 4.4. Histopathology

A microscopic evaluation was performed on two to four larvae pooled from the same experimental run from 11 experimental runs of *C. jejuni*, 10 of *C. upsaliensis*, 12 of *C. helveticus*, three of PBS-inoculated larvae and three of undisturbed control larvae. Evaluations were made during the first three days of the observation period except for one control and one *C. jejuni* run on the 10^th^ day. Due to the severe loss of tissue architecture in most longitudinal sections, only transverse sections were scored and evaluated. There were, on average, 4.4 sections (2.0 standard deviation) per experimental run; all were evaluated and scored. Larvae were placed in neutral-buffered 10% (vol/vol) formalin and fixed for a minimum of two days. Longitudinal and transverse sections were processed routinely into paraffin and 4-µm sections stained with haematoxylin and eosin. The abundance of haemocytes, haemolymph, pigment, nodules, bacteria and adipose tissue (fat body) was scored semi-quantitatively and the assessor was blinded to treatment groups. Haemocytes were scored as one (low number of individual cells), two (low to moderate number of clusters) and three (numerous clusters/sheets), and haemolymph and fat bodies as one (fat body taking ≤ 25% of cross-sections), two (~50% of sections) and three (≥ 75% of sections). Pigment was scored as zero (no pigmentation), one (faint pigmentation at 4× magnification), two (moderate pigmentation at 1.25× magnification) and three (obvious dark pigmentation at 1.25× magnification). Nodules, as aggregations of pigmented haemocytes, were scored as zero (none), one (few/section) and two (over 10/section). Bacteria were scored as zero (none), one (< 25% filling of gut lumen) and two (> 25% of gut lumen) in gut tissue and as presence/absence in haemolymph.

### 4.5. Statistical Analysis

Statistical and exploratory data analyses were performed using R v3.2.2 (R: A language and environment for statistical computing, R Core Team (2013), R Foundation for Statistical Computing, Vienna, Austria. URL: http://www.R-project.org/). An analysis of survival data was performed using log-rank tests of Kaplan–Meier survival curves and Cox proportional hazards mixed regression models. For modelling, the bacterial dose was log_10_ transformed, and its functional form was evaluated by plotting model coefficients against a categorical transformation of the dose and by locally estimated scatterplot smoothing (LOESS) regression of martingale residuals against the dose of models with and without the dose included. With the assumptions being satisfied, the bacterial dose was centred to the mean and used as a continuous covariate. Models were built using the forward step procedure and selected based on ANOVA tests. Kruskal–Wallis non-parametric tests were used for the testing of histopathology scores between the infected and uninfected larvae, and between *Campylobacter* spp. within the infected larvae. Spearman’s rank correlation test was used for the testing of correlations between ordered factors. All statistical analyses used a level of significance at α = 0.05.

## Figures and Tables

**Table 1:** Distribution of histopathology scores * in larvae infected with *Campylobacter* spp. and the uninfected control larvae groups.

Feature	Score	*C. jejuni* (*n* = 11)	*C. upsaliensis* (*n* = 10)	*C. helveticus* (*n* = 12)	PBS-Inoculated (*n* = 3)	Undisturbed Larvae (*n* = 3)
Fat body	1	4	0	1	0	0
	2	3	7	3	0	1
	3	4	3	8	3	2
Haemolymph	1	3	5	8	1	0
	2	7	5	4	2	3
	3	1	0	0	0	0
Haemocytes	1	2	5	3	0	0
	2	6	3	7	3	3
	3	3	2	2	0	0
Nodules	0	1	3	3	3	3
	1	6	3	5	0	0
	2	4	4	4	0	0
Pigment	0	2	2	3	3	3
	1	5	2	0	0	0
	2	3	2	5	0	0
	3	1	4	4	0	0
Bacteria	0	2	3	2	2	1
	1	6	6	9	1	2
	2	2	1	1	0	0

* Haemocytes were scored as one (low number of individual cells), two (low to moderate number of clusters) and three (numerous clusters/sheets), and haemolymph and fat bodies as one (fat body taking ≤ 25% of cross-sections), two (~50% of sections) and three (≥ 75% of sections). Pigment was scored as zero (no pigmentation), one (faint pigmentation at 4× magnification), two (moderate pigmentation at 1.25× magnification) and three (obvious dark pigmentation at 1.25× magnification). Nodules as aggregations of pigmented haemocytes were scored as zero (none), one (few/section) and two (over 10/section). Bacteria were scored as zero (none), one (<25% filling of gut lumen) and two (>25% of gut lumen) in gut tissue and as presence/absence in haemolymph. The scoring of bacteria was unsuccessful in one run of *C. jejuni*-inoculated larvae.

**Table 2:** Details of *Campylobacter* species isolates used in the study.

*C. jejuni*	Source	MLST * Group
NCTC11351	Cattle	ST-605/CC-43
ACP17c	Dog	ST-474/CC-48
ACP50a	Cat	ST-48/CC-48
ACP57b	Dog	ST-45/CC-45
ACP75a	Dog	ST-21/CC-21
ACP90c	Dog	ST-474/CC-48
ACP103c	Dog	ST-45/CC-45
ACP103d	Dog	ST-45/CC-45
ACP117c	Cat	ST-696/CC-1332
ACP176d	Dog	ST-474/CC-48
ACP191a	Dog	ST-61/CC-61
BD12f2b	Dog	ST-3676/CC-42
BD13d3a	Dog	ST-137/CC-45
H450	Human	ST-61/CC-61
H550	Human	ST-42/CC-42
H1763	Human	ST-50/CC-21
H1792	Human	ST-520/CC-21
H1796	Human	ST-45/CC-45
H1799	Human	ST-48/CC-48
H1804	Human	ST-48/CC-48
H1849	Human	ST-137/CC-45
H1878	Human	ST-21/CC-21
H1884	Human	ST-474/CC-48
H1910	Human	ST-21/CC-21
H1924	Human	ST-45/CC-45
H1969	Human	ST-61/CC-61
H1972	Human	ST-42/CC-42
H1978	Human	ST-50/CC-21
H1987	Human	ST-50/CC-21
H22082	Human	ST-474/CC-48
P110b	Poultry	ST-474/CC-48
P525a	Poultry	ST-2381/NA
P836a	Duck	ST-3609/CC-48
P970a	Duck	ST-3961/NA
***C. upsaliensis***		**Source**
ACP5b, ACP18a, ACP19b, ACP64a, ACP72b, ACP144b, ACP136a, ACP149b, BD16e4a, ESR3675		Dogs
ACP9b, ACP135c, ACP170a, ACP170b, ACP179b		Cats
LR128, LR129, LR130, LR131, LR132		Wildlife
ERL103233, ERL112092		Humans
***C. helveticus***		**Source**
ACP102a, ACP105a, ACP105b, ACP108a, ACP110b, ACP114b, ACP123b, ACP175a, ACP183a, CCUG30563, CCUG30682, CCUG30683		Cats
ACP141a		Dog

* Multi-locus sequence typing scheme: sequence type (ST)/clonal complex (CC).

## Supplementary Materials

The following are available online at https://www.mdpi.com/2076-0817/9/9/713/s1, Figure S1: Kaplan–Meier survival curves of larvae inoculated with *Campylobacter* spp. (~10^8^ CFU/mL) and control (phosphate-buffered saline (PBS) inoculated and undisturbed) groups in optimal temperature and atmospheric, and altered incubating conditions according to the suitability for in vitro isolation of *Campylobacter* species. Figure S2. Kaplan–Meier survival curves of larvae inoculated with temperature-inactivated whole-cells and secreted products of *Campylobacter* species.
