# Supplementary material for: Comparison of the Pathogenic Potential of Campylobacter jejuni, C. upsaliensis and C. helveticus and Limitations of Using Larvae of Galleria mellonella as an Infection Model
Source: Pathogens. 2020 Aug 29;9(9):713. doi: 10.3390/pathogens9090713 (PMC7560178; doi:10.3390/pathogens9090713)
Supplement: Supplementary file 1 [file pathogens-09-00713-s001.pdf]

Supplementary Material

Comparison of the Pathogenic Potential of *Campylobacter jejuni*, *C. upsaliensis* and *C. helveticus* and Limitations of Using Larvae of *Galleria mellonella* as an Infection Model

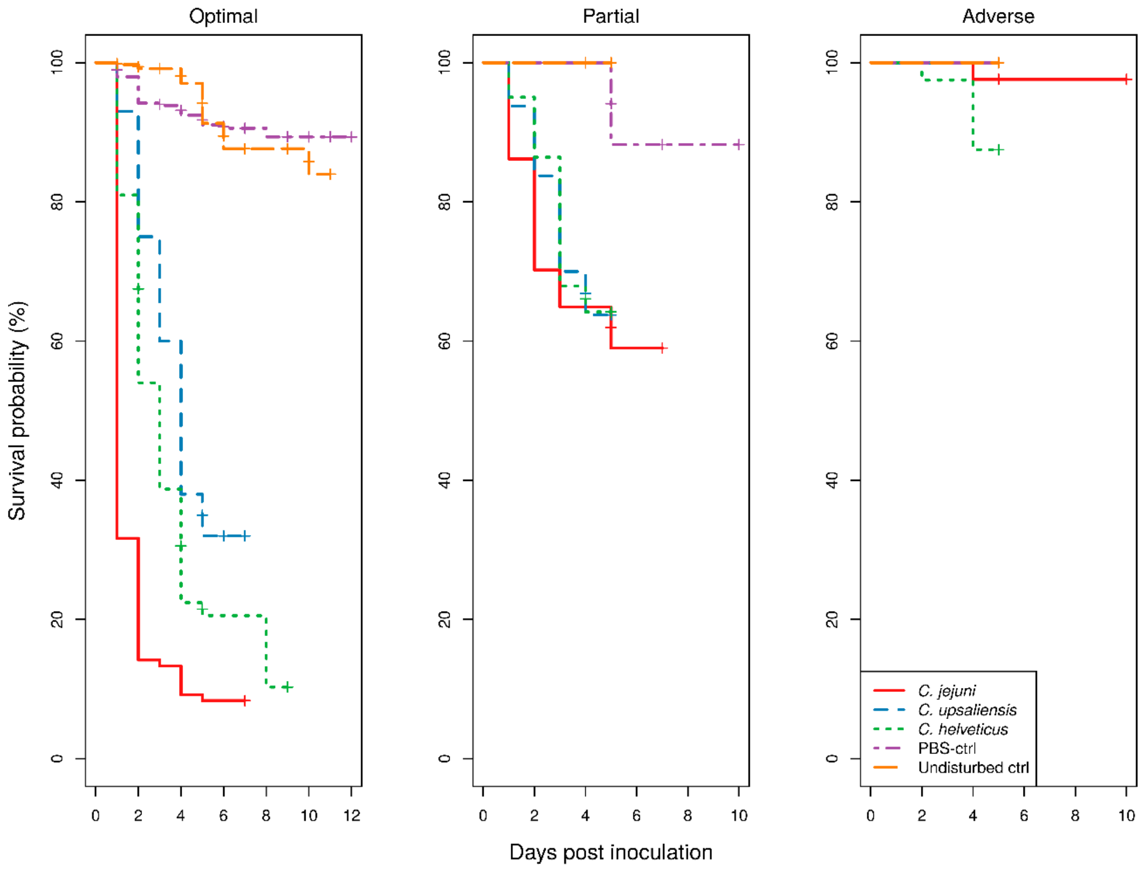

Figure S1. Kaplan-Meier survival curves of larvae inoculated with *Campylobacter* spp. (~10^8 CFU/mL) and control (phosphate-buffered saline (PBS) inoculated and undisturbed) groups in optimal temperature and atmospheric, and altered incubating conditions according to the suitability for *in vitro* isolation of *Campylobacter* species. "Optimal" denotes an H_2-enriched microaerobic atmosphere at 37°C, "Adverse" an ambient room atmosphere and temperature while "Partial" denotes an alteration of only temperature or atmosphere from optimal to adverse conditions. The survival curves of larvae inoculated with *Campylobacter* spp. in the "Partial" and "Adverse" environments are summations of two to four strains of each species tested in technical duplicates while the data in the "Optimal" environment is a summation of the same strains tested with the corresponding bacterial load and included two to four biological replicates each with technical duplicates. Experimental runs consisted of sets of 10-15 larvae and resulted in 302 larvae in "Partial" environment (94 *C. jejuni*, 80 *C. upsaliensis*, 81 *C. helveticus*, 27 PBS-inoculated and 20 undisturbed), 142 larvae (42 *C. jejuni*, 40 each of *C. upsaliensis* and *C. helveticus*, and 10 each of PBS-inoculated and undisturbed) in "Adverse" environmental conditions, and 360 larvae (120 *C. jejuni* and 100 each of *C. upsaliensis* and *C. helveticus*, and 20 each of PBS-inoculated and 20 undisturbed) in "Optimal" environment.

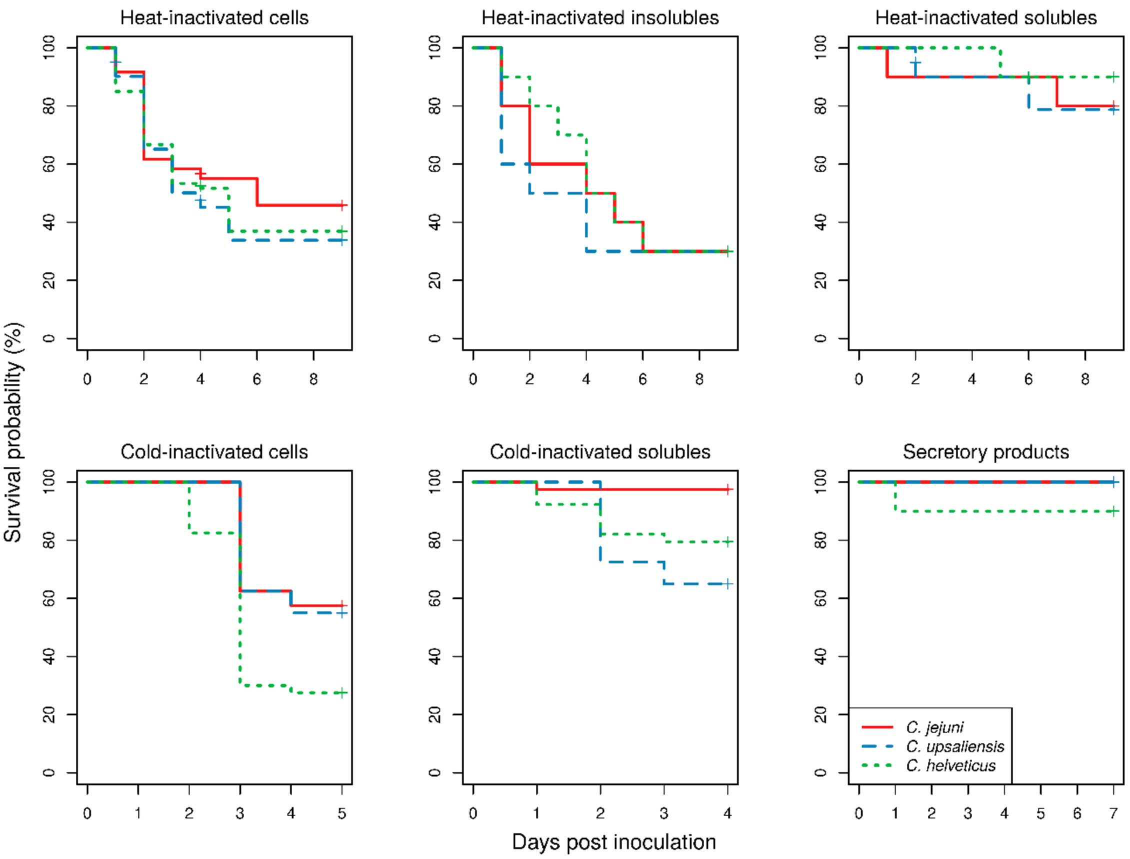

Figure S2. Kaplan-Meier survival curves of larvae inoculated with temperature-inactivated whole-cells and secreted products of *Campylobacter* species. The survival curves of larvae inoculated with bacterial material are summations of two to four strains of each species tested in technical duplicates in sets of 10-15 larvae. In total, 170 larvae were tested for each of the species. The inocula for temperature-inactivated assays were prepared at ~10^8 CFU/mL. The heat-inactivation of bacteria was performed by heating the inocula at 100°C for 10 minutes and used as a heat-inactivated cell assay and was further separated by centrifugation at 12,000 g for 3 minutes into soluble (supernatant) and insoluble (pelleted cells resuspended in one mL of phosphate-buffered saline) components. The cold-inactivation of bacteria was performed by a triple freeze-thaw procedure between -80°C and 42°C in three cycles of 30 minutes duration and used as suspensions of cold-inactivated cell assay and further separated into soluble and insoluble components as for the heat-inactivation assays. The cold-inactivated insoluble assay was omitted due to a visible inhomogeneity of samples that could not be improved by vortexing. For the heat- and cold-inactivated assays the inactivation was checked by culture. For the secretory products assay, bacteria were subcultured into 6 mL of Mueller-Hinton broth in air-tight boxes at 200 rotations per minute until turbid (usually two days for *C. jejuni* and *C. upsaliensis* and four days for *C. helveticus*). The broth was centrifuged as for the heat-inactivation assay with the supernatant used as the secretory products assay. One control larvae group was injected with sterile Mueller-Hinton broth or phosphate-buffered saline and the other group was left undisturbed but as no deaths occurred the data is omitted from the plot. All assays were done in H_2-enriched microaerobic atmosphere at 37°C.
